# Favorable Outcome of Nasal-Type Extranodal Natural Killer (NK)/T-Cell Lymphoma in a Very Elderly Patient Treated With Radiotherapy Alone

**DOI:** 10.7759/cureus.89939

**Published:** 2025-08-12

**Authors:** Natsumi Hara, Natsuko Saito-Sasaki, Etsuko Okada, Yu Sawada

**Affiliations:** 1 Dermatology, University of Occupational and Environmental Health, Kitakyushu, JPN

**Keywords:** case report, elderly, lymphoma, nasal-type extranodal nk/t-cell lymphoma, radiation

## Abstract

Extranodal natural killer (NK)/T-cell lymphoma, nasal type (ENKTCL), is a rare and aggressive Epstein-Barr virus *(*EBV)-associated malignancy, typically affecting middle-aged individuals. Cases in patients over 80 are extremely rare. We report an 88-year-old woman, the oldest known case, successfully treated with radiotherapy alone. She presented with erythema and swelling of the right cheek and nasal cavity, initially misdiagnosed as facial cellulitis. A skin biopsy revealed atypical CD3⁺/CD56⁺ lymphoid cells, and EBER positivity confirmed EBV involvement. Imaging showed localized disease without systemic spread. Due to her advanced age and comorbidities, chemotherapy was not feasible, and she received radiotherapy alone, resulting in complete remission without recurrence. This case highlights the effectiveness of radiotherapy in localized ENKTCL and suggests that even very elderly patients can achieve favorable outcomes with appropriate treatment.

## Introduction

Extranodal natural killer (NK)/T-cell lymphoma, nasal type (ENKTCL), is a rare and aggressive malignancy most often seen in East Asian and Latin American populations [1]. It arises most commonly in the nasal cavity or upper aerodigestive tract, but its clinical presentation can be variable [2]. The disease is often characterized by rapid progression, angioinvasion, and tissue necrosis, and prognosis is generally poor, especially in advanced stages or in patients with systemic involvement [3]. Here, we report the case of an 88-year-old woman diagnosed with localized nasal-type ENKTCL, successfully treated with radiotherapy alone. This represents the oldest known case reported in the literature and illustrates the importance of clinical suspicion, accurate histopathological diagnosis, and individualized treatment planning in elderly patients. 

This article was previously presented at the 398th Fukuoka Regional Meeting of the Japanese Dermatological Association on September 12, 2021.

## Case presentation

**Figure 1:**
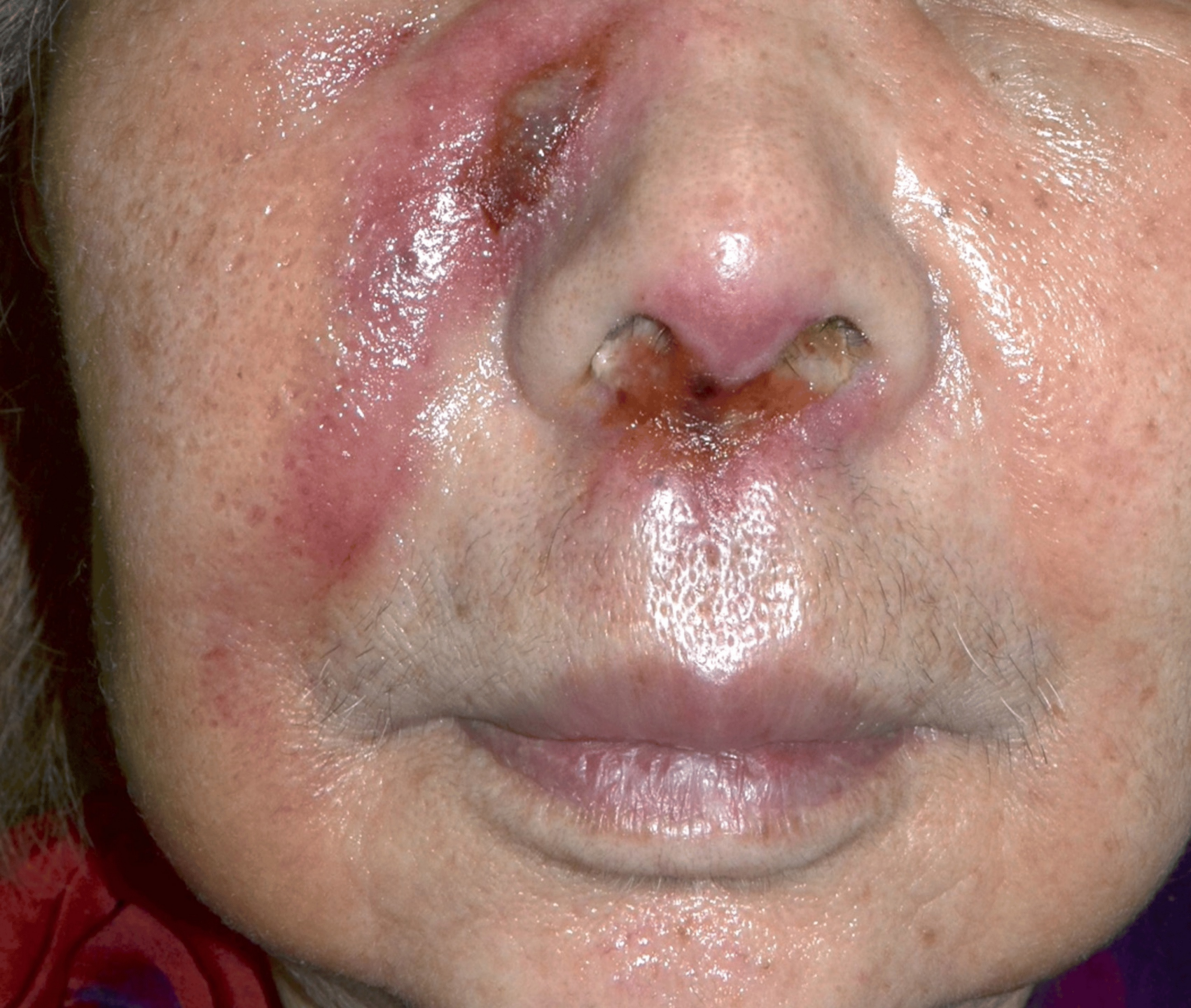
Clinical manifestation Erythema and swelling on the right cheek and nasal area.

**Figure 2:**
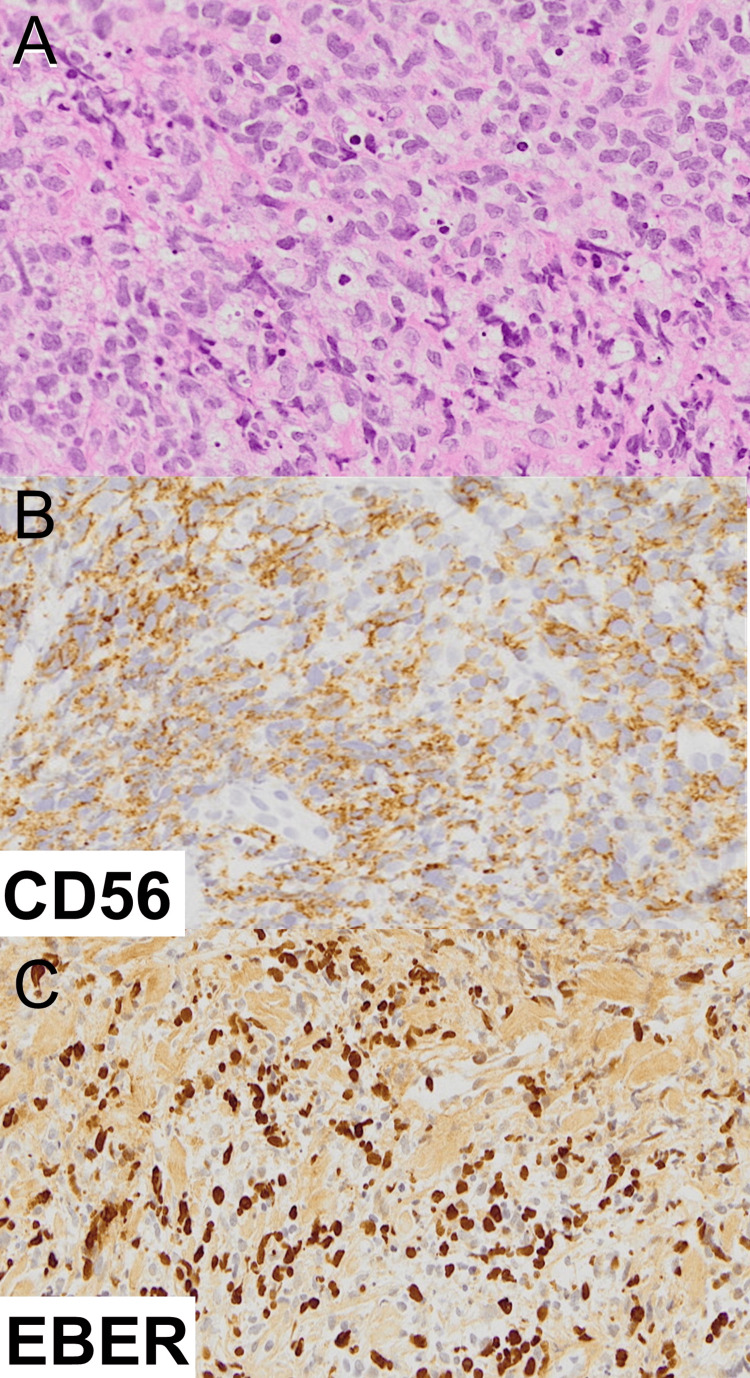
Histological examination (A) Skin biopsy showing dense infiltration of atypical lymphoid cells in the dermis. (B) Immunohistochemical staining showing positivity for CD56. (C) In situ hybridization for EBV-encoded RNA (EBER) showing nuclear positivity in tumor cells.

**Figure 3:**
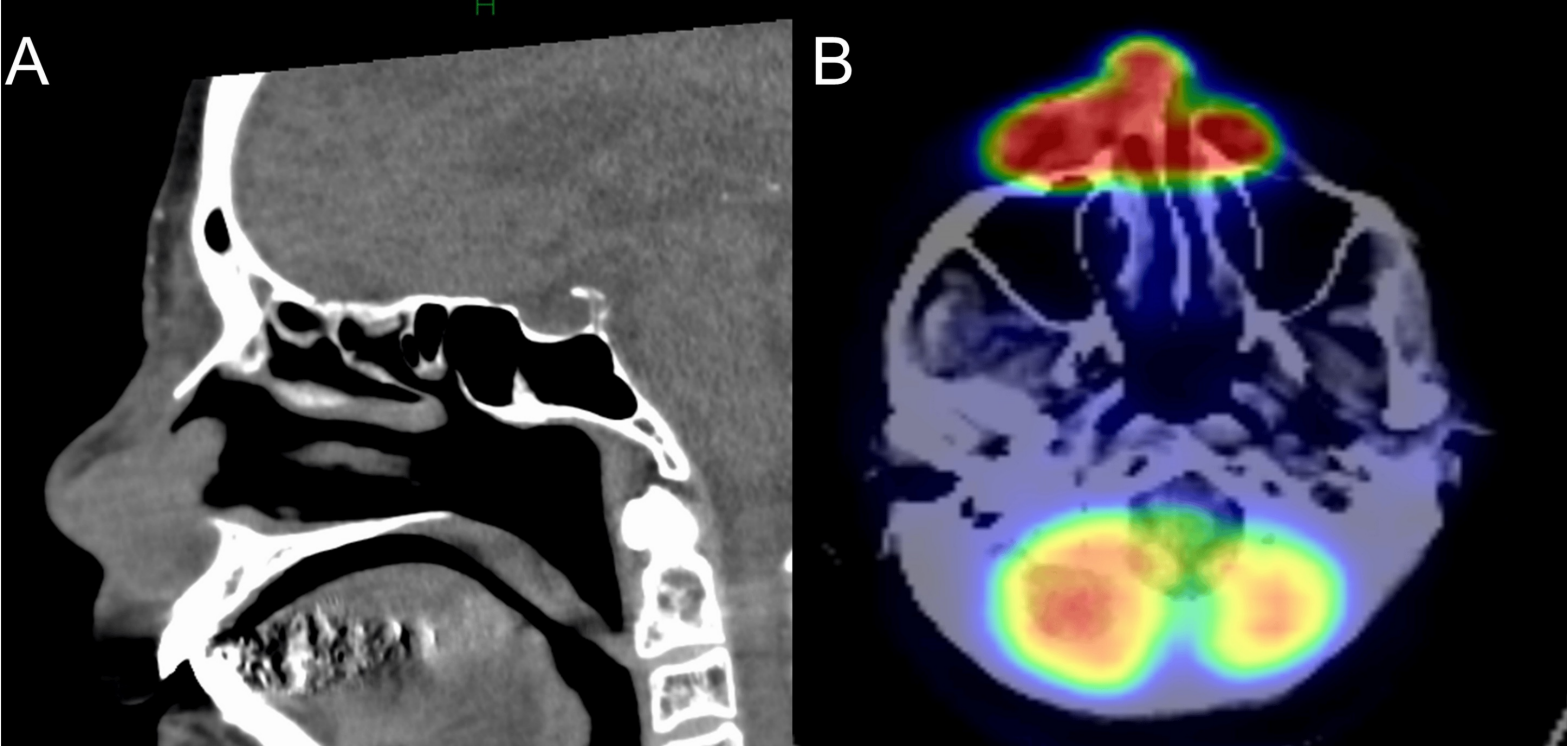
CT and PET-CT findings (A) CT scan showing skin thickening and increased density of the subcutaneous fat tissue in the nasal region. (B) PET-CT showing abnormal FDG uptake in the nasal and perinasal areas, with no evidence of systemic involvement.

An 88-year-old woman presented with erythema and swelling on her right cheek and nasal cavity. She was initially diagnosed with facial cellulitis at a local dermatology clinic and treated with antibiotics, but showed little improvement. She was then referred to our department for further evaluation. On examination, diffuse erythema and swelling were noted on the right cheek extending to the nasal cavity (Figure 1). Laboratory tests revealed mildly elevated levels of lactate dehydrogenase (LDH: 270 U/L; normal range: 124-222 U/L) and soluble interleukin-2 receptor (sIL-2R: 996 U/mL; normal range: 122-496 U/mL). A skin biopsy showed infiltration of atypical lymphoid cells positive for CD3 and CD56, and EBV-encoded RNA (EBER) in situ hybridization confirmed EBV infection (Figures 2A-2C). CT imaging revealed skin thickening and subcutaneous fat stranding from the right upper eyelid to the cheek. PET-CT demonstrated abnormal FDG uptake in the right cheek and nasal area, with no evidence of systemic involvement (Figures 3A-3B). These findings supported a diagnosis of localized nasal-type extranodal NK/T-cell lymphoma. Given her advanced age and comorbidities, including complete atrioventricular block with pacemaker and carotid artery stenosis, systemic chemotherapy was deemed unsuitable. She was treated with localized radiotherapy (44 Gy/20 Fr to the nasal cavity and 20 Gy/5 Fr to the cervical region), achieving excellent local control with no recurrence during follow-up.

## Discussion

To date, the oldest documented case of nasal-type ENKTCL in the literature was 83 years old [4]. Our patient, at 88 years of age, represents the oldest reported case to date, highlighting the importance of recognizing this rare yet aggressive malignancy even in the very elderly. While ENKTCL is known for its aggressive clinical course, angioinvasion, and high mortality, especially in advanced stages, early recognition and appropriate localized treatment can yield favorable outcomes.

Dermatologists may play a pivotal role in the early diagnosis of ENKTCL, especially when initial manifestations are cutaneous or localized facial swelling is misdiagnosed as cellulitis, as occurred in our case. Awareness of such atypical presentations is crucial, particularly in elderly individuals, where nonspecific symptoms are often attributed to more common benign conditions. Prompt skin biopsy and appropriate immunophenotyping can lead to an accurate diagnosis and timely initiation of treatment.

Interestingly, despite the patient’s advanced age and multiple comorbidities, radiotherapy alone led to excellent disease control without significant toxicity. ENKTCL is known to be highly radiosensitive, and this case reinforces the growing body of evidence that radiotherapy is not only an effective modality but may also be a preferable choice for elderly or frail patients for whom chemotherapy is not feasible due to potential toxicity or intolerance. In a large retrospective study involving 321 patients with early-stage ENKTCL, radiotherapy alone was associated with a five-year overall survival (OS) rate of 61.2% and progression-free survival (PFS) of 56.4%, both significantly superior to outcomes seen with chemotherapy alone [5].

Additionally, data suggest that radiotherapy may achieve curative potential in early-stage low-risk patients. According to the Prognostic Index for Natural Killer cell lymphoma (PINK) (6), our patient scored 1 point based solely on age over 60, classifying her as low risk. This classification aligns with her favorable clinical course. The absence of systemic disease, localized presentation, and potential age-related changes in tumor biology - such as lower proliferative indices or increased radiosensitivity - may have contributed to the observed treatment success. Notably, studies have reported that low-risk patients treated with radiotherapy exhibit survival outcomes comparable to age-matched controls in the general population [5].

Another consideration is the evolving understanding of the tumor microenvironment and immunosenescence in elderly patients. While aging is often associated with impaired immune surveillance, it may paradoxically alter tumor immunogenicity or angiogenic profiles, rendering certain lymphomas more amenable to local control. Furthermore, in older individuals, reduced expression of proliferation markers such as Ki-67 and lower EBV-DNA titers have been observed in some ENKTCL cases, potentially correlating with a less aggressive disease phenotype.

According to the PINK [6], our patient scored 1 point (age >60), placing her in the low-risk group. The favorable outcome may reflect the limited disease extent, lack of systemic involvement, and age-related tumor biology such as lower proliferative activity or increased radiosensitivity. These factors may explain the effectiveness of radiotherapy alone in this very elderly patient.

## Conclusions

This case illustrates that, even in very elderly patients with comorbidities, early-stage nasal-type ENKTCL can be effectively treated with radiotherapy alone. Given the tumor’s radiosensitivity and the risks associated with systemic chemotherapy in older adults, individualized treatment approaches are essential. Clinicians should maintain a high index of suspicion when evaluating persistent facial swelling, especially in populations at risk, and recognize the critical role of histopathological diagnosis. Our report adds to the growing evidence that radiotherapy remains a viable and potentially curative option for localized ENKTCL, even in patients beyond 80 years of age.
